# The Prize Winners with Selected Papers

**Published:** 1918-12-14

**Authors:** 


### "THE HOSPITAL" LEAFLET COMPETITION.

### The Prize Winners with Selected Papers.

IN our issue of August 17 we ventured to state that in our view many of the authorities of those hospitals which have suffered most from the shortage of labour, have not set to work in the right way to secure probationers. Our suggestion was that where there is a shortage the authorities should develop their needs locally, and the first step to success will be found in an attractive leaflet setting forth the conditions of service, its material attractions, and its moral claims on the community. Nearly fifty years ago, in association with the late Mrs. Wardroper, we adopted this course at a time when the prejudice against hospitals was so great that it was well-nigh impossible to recruit respectable and educated women for the work of nursing. Our appeals for probationers at that time were directed to certain definite types of the community, and it was this system, pursued by advertisement in the provincial papers, mainly circulating in the agricultural districts and smaller towns, that caused the people to begin to take an interest in the Nursing Service. From that date forward that interest has continued, and in recent years has developed by leaps and bounds.

It gives us great pleasure to report the most gratifying success which has attended our request to the readers of THE HOSPITAL for leaflets on branches of the Nursing Service. As an encouragement to them to set forth certain conditions (see THE HOSPITAL, August 17, 1918, page 438), three prizes were offered of £3 3s., £2 2s., and £1 1s. respectively. The judges have considered the work of two of the competitors as equal, and the third prize of £1 1s. is divided between them accordingly.

